# Is Financial Hardship Associated with Reduced Health in Disability? The Case of Spinal Cord Injury in Switzerland

**DOI:** 10.1371/journal.pone.0090130

**Published:** 2014-02-28

**Authors:** Christine Fekete, Johannes Siegrist, Jan D. Reinhardt, Martin W. G. Brinkhof, for the SwiSCI Study Group

**Affiliations:** 1 Swiss Paraplegic Research, Nottwil, Switzerland; 2 Senior Professorship for Work Stress Research, Faculty of Medicine, University of Duesseldorf, Life Science Center, Duesseldorf, Germany; 3 Institute for Disaster Management and Reconstruction, Sichuan University and Hong Kong Polytechnic University, Chengdu, Sichuan Province, China; 4 Department of Health Sciences and Health Policy, University of Lucerne, Lucerne, Switzerland; CUNY, United States of America

## Abstract

**Objective:**

To investigate socioeconomic inequalities in a comprehensive set of health indicators among persons with spinal cord injury in a wealthy country, Switzerland.

**Methods:**

Observational cross-sectional data from 1549 participants of the Swiss Spinal Cord Injury Cohort Study (SwiSCI), aged over 16 years, and living in Switzerland were analyzed. Socioeconomic circumstances were operationalized by years of formal education, net equivalent household income and financial hardship. Health indicators including secondary conditions, comorbidities, pain, mental health, participation and quality of life were used as outcomes. Associations between socioeconomic circumstances and health indicators were evaluated using ordinal regressions.

**Results:**

Financial hardship was consistently associated with more secondary conditions (OR 3.37, 95% CI 2.18–5.21), comorbidities (OR 2.88, 95% CI 1.83–4.53) and pain (OR 3.32, 95% CI 2.21–4.99), whereas mental health (OR 0.23, 95% CI 0.15–0.36), participation (OR 0.30, 95% CI 0.21–0.43) and quality of life (OR 0.22, 95% CI 0.15–0.33) were reduced. Persons with higher education reported better mental health (OR 1.04, 95% CI 1.00–1.07) and higher quality of life (OR 1.06, 95% CI 1.02–1.09); other health indicators were not associated with education. Household income was not related to any of the studied health indicators when models were controlled for financial hardship.

**Conclusions:**

Suffering from financial hardship goes along with significant reductions in physical health, functioning and quality of life, even in a wealthy country with comprehensive social and health policies.

## Introduction

An inverse association of socioeconomic circumstances with various health indicators is one of the most robust findings of social-epidemiological research [1]–[3]. Major evidence comes from cohort studies of general populations, whereas relatively few investigated social inequalities in health among persons with disabilities [4]–[11]. Given an increasing proportion of persons with disabilities in rapidly ageing societies, this gap in knowledge needs to be addressed, as detailed information on social determinants of health among different population groups is required to inform targeted developments of social and health policies.

Previous studies on health inequalities in persons with disabilities have addressed the concept of ‘health’ by using particular health indicators such as functional limitations [4]–[6], [8], [9], pain [5], [10], depression [6], mental disorders [10], or quality of life [11]. Yet, a comprehensive assessment of health based on a combination of indicators is widely lacking [6], [10]. Furthermore, most studies on health inequalities among persons with disabilities used established individual-level indicators of socioeconomic circumstances (i.e., education [4], [5], [7], [9], income [4], occupational status [5], [9]) or macro-level indicators such as neighborhood indices [8], [10], [11]. While these indicators mirror peoples' chronic and more distant social standing, more proximal and contemporary socioeconomic circumstances such as the perception of financial hardship may more accurately reflect their lived experience. Preliminary evidence in persons with disabilities [8] or general populations [12], [13] indicates that financial hardship may limit functioning and health.

In this study, we set out to tackle these shortcomings by investigating social inequalities in health among adults with a severe physical impairment, namely spinal cord injury (SCI). SCI has a far-reaching impact on a person's functioning and health as affected persons suffer from a loss of sensory and motor function below the lesion level [14]. So far, one research team has studied health inequalities in SCI using data from the SCI Model System program in the USA, using education and income as established indicators for socioeconomic circumstances to predict different health outcomes [15]–[20]. As the study population essentially included U.S. residents with health care insurance only [15]–[20], results may not be representative for all U.S. people living with SCI [15]–[20]. We extend and enrich this research by (1) adding financial hardship as a key indicator of proximal socioeconomic disadvantage; (2) studying a comprehensive set of health indicators based on the framework of the International Classification of Functioning, Disability and Health (ICF) [21], and (3) using a population-based sample. Importantly, our analysis presents an informative case regarding social inequalities in health, because subjects were recruited in Switzerland, one of the wealthiest countries worldwide with highly developed health care systems and extended social security policies [22], [23]. If health inequalities are observed in Switzerland we then would expect them to be even more pronounced in less wealthy countries [23].

The aim of this study is to investigate social inequalities in a comprehensive set of health indicators in persons with SCI. We therefore analyze the association between socioeconomic circumstances (years of formal education, net equivalent household income, financial hardship) and various health indicators including secondary conditions, comorbidities, pain, mental health, participation, and quality of life.

## Methods

### Sample

We analyzed cross-sectional data from the population-based community survey of the Swiss Spinal Cord Injury Cohort Study (SwiSCI) [24]. The survey was conducted between late 2011 and early 2013 and included Swiss residents with a traumatic or non-traumatic SCI aged over 16 years. Persons with congenital conditions leading to SCI, with new SCI in the context of palliative care, with neurodegenerative disorders, and with Guillain-Barré syndrome were excluded from the study. The SwiSCI population was recruited through the national association for persons with SCI (Swiss Paraplegic Association), three specialized SCI-rehabilitation centers, and a SCI-specific home care institution [24]. The survey featured written or online questionnaires, and in special cases, telephone interviews. This study has been approved by the Medical Ethical Committee of the Canton Lucerne, Switzerland. In addition, the study protocol has been approved by the Steering Committee of the SwiSCI study. All participants have signed a written consent form.

### Measures

*Socioeconomic circumstances*: Level of education, net equivalence household income and perceived financial hardship were chosen as three indicators of individual-level socioeconomic circumstances. Education was classified according to the International Standard Classification of Education as total years of formal education, combining school and vocational training [25]. For bivariate analyses, education was regrouped in four categories according to the Swiss National Cohort [26]. In multivariable models, education was introduced continuously. Income was measured by net equivalent household income in Swiss Francs, including information on disposable income, household size and number of adults and children according to the Organisation for Economic Co-operation and Development (OECD) criteria [27]. Income was reclassified into distribution-based quartiles for bivariate analysis and entered into multivariable models as continuous variable. Income was divided by 1000 in order to receive legible effect sizes in multivariable models. Financial hardship was assessed by the question ‘Did you experience financial difficulties that restricted your everyday life (participation) over the past four weeks?’. Answer categories were ‘not applicable’, ‘had no impact’, ‘has complicated my life somewhat’, and ‘has complicated my life massively’. By combining the first two categories, a three-categorical variable was computed.

*Health conditions* & *body functions*: To assess the prevalence of health problems in addition to SCI, a sum score of four dichotomous items on comorbidities (diabetes, heart disease, cancer, depression) based on the Self-Administered Comorbidity Questionnaire [28] was computed (range 0–4, higher scores indicating more comorbidities). Due to a low prevalence of persons indicating more than two comorbidities, the variable was classified into ‘having no comorbidities’, ‘having one comorbidity’, and ‘having two or more comorbidities’ for multivariable analysis. Information on the prevalence and severity of secondary conditions during the past three months was collected using the Spinal Cord Injury Secondary Conditions Scale (SCI-SCS) [29]. The SCI-SCS measures 16 SCI-relevant conditions by a 4-point Likert scale (2 items on pain and 1 item on diabetes were not assessed by SCI-SCS in this study as respective information was gathered in other questions). The SCI-SCS therefore contains 13 items and ranges from 0–39, higher scores indicating higher prevalence and severity. Referring to ICF Domains [21], 4 items are classified as health conditions (contractures, urinary tract infections, injury caused by loss of sensation, pressure sores), 6 items as body functions (sexual dysfunction, spasticity, bladder dysfunction, bowel dysfunction, autonomic dysreflexia, postural hypertension), and 3 items can be assigned to both categories (circulatory problems, respiratory problems, heterotopic ossification). Secondary conditions were classified into distribution-based quartiles (0–5; 6–10; 11–14; ≥15) for multivariable analysis (see section on Statistical analysis). Pain intensity (body function) was assessed using a ten-point Likert-scale [30]. This information has been categorized into ‘no pain’, ‘mild (1–3)’, ‘moderate (4–6)’ and ‘severe (>6)’ pain [31] for multivariable analysis. To measure mental health (body function), we used the 5-item Mental Health Index of the 36-item Short Form Health Survey (SF-36) and computed a sum score ranging from 0-100 with higher scores indicating better mental health [32]. For multivariable analysis, distribution-based quartiles were computed (0–60; 61–76; 77–84; ≥84).

*Activity *& *participation* was measured using the 11-item subscale on participation restrictions of the Utrecht Scale for Evaluation of Rehabilitation-Participation (USER-participation) [33]–[35] which employs a 4-point Likert type rating scale. A sum score of the ratings of items that were applicable to a person was computed and converted into a score ranging from 0–100. The higher the score, the lower the participation restrictions [33]. The score has been categorized into distribution-based quartiles for multivariable analysis (0–54.9; 55–72.9; 73–87.9; ≥88).

*Quality of life* was assessed by a single item rated on a 5-point Likert-scale ranging from 0 (‘very poor’) to 4 (‘very good’) [36]. Due to a low prevalence of ‘very poor’ quality of life, the categories ‘very poor’ and ‘poor’ were combined for multivariable analysis.

*Control variables*: Age, gender, lesion characteristics (years since injury, para- vs. tetraplegia, complete vs. incomplete lesion), aetiology (traumatic vs. non-traumatic), and social support were included as potential confounders in multivariable analyses. Severity of disability had been linked to diverse health indicators [37] and might be associated with downward social mobility, leaving those with higher lesion level and complete lesions at higher risk. Aetiology is an important potential confounder in associations with social inequalities of health as persons with traumatic SCI are privileged in the Swiss insurance system. Given its relevance for health inequalities [38], the degree of social support of SCI persons was introduced as confounder in multivariable models. Social support was assessed by asking about having a partner (‘yes’/’no’) and living arrangement (‘living alone’/’living with others’). Poor social support was coded if the two answers were negative.

### Statistical analyses

Following descriptive analysis of the study population, we explored unadjusted bivariate associations between socioeconomic circumstances and health indicators. Given the fact that dependent variables were not normally distributed, we used a nonparametric ANOVA to evaluate differences between groups (Kruskal-Wallis) and a test for trend across ordered groups according to Cuzick [39].

Next, a set of regression analyses was applied to evaluate associations. As effect sizes of ordinal regressions are easier to interpret as compared to models for continuous dependent variables, ordinal logistic regression models were applied for all outcomes. Ordinal logistic regressions are extensions of logistic regressions [40], but allow to examine the dependence of a polytomous ordinal outcome on several independent variables without losing information through outcome dichotomization. As a prerequisite to apply ordinal logistic regressions, the parallel lines assumption indicating that betas are the same for each transition from an ordinal scale point must be confirmed [41]. This assumption, tested by Stata's gologit2 command and its autofit option, was confirmed in all cases. For education and income, two subsequent series of ordinal logistic models were computed. The first models were adjusted for age, gender, lesion characteristics, aetiology, social support, income and education. To assess the impact of financial hardship on the associations between education, income and health indicators, financial hardship was introduced separately in a second model. For financial hardship, only one model (adjusted for all control variables) was computed. Control variables were mean centered in all analyses.

In the main data analyses, we adjusted for both unit and item nonresponse [42]. Unit nonresponse, which refers to the complete absence of a response by an eligible person of the initial survey population, was 50.7%. Unit nonresponse was accounted for by using inverse probability weights (IPWs) as sampling weights in regression analyses. IPWs were derived from propensity scores in multivariable-adjusted logistic regression analyses. The propensity to participate in the survey was higher for members of the Swiss Paraplegic Association as compared to non-members; convexly shaped with increasing age (highest at age 52); concavely shaped with time since injury (least at 16 years post SCI); but not related to other variables including age, gender, lesion level (paraplegia vs. tetraplegia), and language (including German, French, and Italian). The average IPW used in weighted analyses was 2.03 (range: 1.02–6.65) [42].

Item nonresponse, which refers to the failure of survey respondents to answer a specific question, was addressed using multiple imputation (MI). We used MI by chained equations (MICE)[43] to impute different types of variables, including categorical, ordinal and linear variables. We imputed predictors (socioeconomic circumstances) and control variables, but not outcomes (health indicators). For each model, imputations were carried out for 10 datasets (see Appendix S1 for more detail on MI modeling). In the respective table, odds ratios, 95% confidence intervals, and p values from the conditional (equal fraction-missing-information, FMI) test are presented [44]. The equal FMI test is a likelihood ratio test suitable for multiply imputed datasets.

To assess the risk of bias due to list-wise exclusion of cases with missing data in health indicators, a sensitivity analysis was performed comparing four scenarios:

1. imputed data for socioeconomic circumstances/controls, but only full cases in health indicators (as displayed in the main results),
2. only full cases in all variables,
3. imputed data for socioeconomic circumstances/controls and replacing missings in health indicators by ‘best case’ (i.e. absence of complication, highest quality of life),
4. imputed data for socioeconomic circumstances/controls and replacing missings in health indicators by ‘worst case’ (i.e. presence of comorbidity, lowest quality of life).

Results of the sensitivity analysis are displayed in Table S1 in the Supporting Information. Our findings from scenario 1 were robust and mainly confirmed by the sensitivity analysis. Analyses were conducted using STATA Version 13 for Windows (College Station, TX, USA).

## Results

Sample characteristics are displayed in Table 1. The majority of the sample were males, mean age was 52.3 years. Around one out of five persons was classified as socially low integrated. Almost 70% of the participants had a paraplegia, less than half indicated having a complete lesion and in around four out of five persons, the SCI was caused by a traumatic event. Many persons in our sample live a long time with SCI as mean time since injury was 17 years. Mean education was 13.6 years, with nearly half of the sample being classified in the category ‘secondary education’. Mean net equivalence income was somewhat higher than 4000 Swiss Francs and around 30% of the sample indicated having at least some financial difficulties. On a secondary conditions scale ranging from 0***–***39, persons indicated on average 10.6 points. Nearly three out of four persons did not report a comorbidity and the overall pain intensity was somewhat over 3 in the total sample. On a total score from 0***–***100, mental health scores were on average around 72 and participation around 70, with higher scores indicating better mental health and less participation restrictions. Nearly 60% of the total population rated their quality of life as at least good.

**Table 1:** Basic characteristics of the SwiSCI study population.

[Missing values]	N (%) or mean (SD); median (IQR)
Total	1549 (100)
*Sociodemographic characteristics*	
Male gender	1107 (71.5)
Age in years	52.3 (14.8); 52 (42–63)
Low social support [24]	319 (20.9)
*Lesion characteristics*	
Paraplegia [12]	1063 (69.2)
Complete lesion [9]	646 (42.0)
Traumatic aetiology [15]	1202 (78.4)
Years since injury [38]	17.0 (12.6); 14 (6–25)
*Education* [32]	
Years of education	13.6 (3.3); 13 (12–15)
Compulsory schooling (≤9 years)	143 (9.4)
Vocational training (10–12 years)	377 (24.9)
Secondary education (13–16 years)	721 (47.5)
University education (≥17 years)	276 (18.2)
	
*Net equivalence household income* [168]	4135.7 (1809.6); 3750 (2500–5250)
Lowest quartile (≤2500 Swiss Francs)	359 (26.0)
2^nd^ lowest quartile (>2500–3750)	379 (27.4)
2^nd^ highest quartile (>3750–5250)	314 (22.7)
Highest quartile (>5250)	329 (23.8)
*Financial hardship* [70]	
Severe financial difficulties	128 (8.7)
Some financial difficulties	320 (21.6)
No financial difficulties or not applicable	1031 (69.7)
*Health indicators*	
Secondary conditions (score 0–39) [359]	10.6 (6.4); 10 (6–14)
Comorbidities (score 0–4) [89]	0.33 (0.58); 0 (0–1)
No comorbidities	1065 (73.0)
One comorbidity	311 (21.3)
Two to four comorbidities	84 (5.8)
Pain intensity (scale 0–9) [68]	3.3 (2.8); 3 (0–6)
No pain	476 (32.1)
Mild pain (1–3)	294 (19.9)
Moderate pain (4–6)	301 (20.3)
Severe pain (>6)	410 (27.7)
Mental health (score 0–100) [187]	72.2 (17.6); 76 (60–84)
Participation (score 0–100) [58]	70.0 (21.7); 72.7 (54.5–87.9)
Quality of life (score 0–4) [50]	2.6 (0.9); 3 (2–3)
Very poor	29 (1.9)
Poor	118 (7.9)
Neither poor nor good	459 (30.6)
Good	704 (47.0)
Very good	189 (12.6)

Abbreviations: SD standard deviation, IQR interquartile range.

Results from bivariate analysis are displayed in Table 2. Associations with education and income were statistically significant for five out of six health indicators (comorbidities, pain, mental health, participation, and quality of life). For all health indicators, significant and graded associations with financial hardship were apparent.

**Table 2:** Socioeconomic circumstances and health indicators, mean and (standard deviation).

	Secondary conditions^a^	Comorbidities^a^	Pain intensity^a^	Mental health^b^	Participation^b^	Quality of life^b^
Score	0–39	0–4	0–9	0–100	0–100	0–4
**Education in years**						
Compulsory schooling	11.90 (7.36)	0.55 (0.69)	3.66 (2.91)	63.26 (21.02)	59.03 (25.68)	2.24 (0.85)
Vocational training	10.87 (6.80)	0.33 (0.58)	3.48 (2.83)	72.54 (17.67)	69.28 (21.97)	2.58 (0.86)
Secondary education	10.21 (6.26)	0.30 (0.55)	3.16 (2.74)	73.83 (16.65)	72.03 (20.93)	2.66 (0.88)
University education	10.86 (5.92)	0.29 (0.55)	3.05 (2.78)	71.94 (17.21)	72.18 (19.44)	2.69 (0.86)
p value^c^	0.066	<0.001	0.077	<0.001	<0.001	<0.001
p value^d^	0.468	0.001	0.012	0.004	<0.001	<0.001
**Net equivalence income (quartiles)**						
Lowest	11.14 (6.70)	0.41 (0.64)	3.74 (2.83)	68.49 (19.12)	68.06 (22.51)	2.42 (0.83)
2^nd^ lowest	10.63 (6.32)	0.31 (0.54)	3.23 (2.76)	71.72 (17.71)	70.06 (21.42)	2.57 (0.88)
2^nd^ highest	10.31 (6.09)	0.27 (0.54)	3.27 (2.74)	72.22 (17.40)	72.49 (19.55)	2.68 (0.84)
Highest	10.31 (6.38)	0.29 (0.55)	2.80 (2.76)	75.85 (15.50)	71.82 (21.79)	2.79 (0.88)
p value^c^	0.411	0.008	<0.001	<0.001	0.076	<0.001
p value^d^	0.108	0.002	<0.001	<0.001	0.013	<0.001
**Financial hardship**						
Severe difficulties	14.21 (6.90)	0.60 (0.74)	4.86 (2.81)	59.14 (21.26)	60.62 (19.06)	2.03 (0.86)
Some difficulties	12.18 (6.53)	0.42 (0.62)	3.70 (2.75)	67.25 (17.37)	67.36 (20.77)	2.36 (0.86)
No difficulties	9.76 (6.12)	0.27 (0.53)	2.96 (2.72)	75.29 (16.07)	72.51 (21.48)	2.75 (0.84)
p value^c^	<0.001	<0.001	<0.001	<0.001	<0.001	<0.001
p value^d^	<0.001	<0.001	<0.001	<0.001	<0.001	<0.001

a Higher scores indicate more secondary conditions, more comorbidities and higher pain intensity.

b Higher scores indicate better mental health, less participation restrictions, and higher quality of life.

c p values from Kruskal-Wallis tests (adjusted for ties).

d p values from test for trend across ordered groups.

Note: only full cases and unweighted results in this Table.

Results from multivariable analysis are presented in Table 3. After controlling for potential confounders, higher education was significantly related to better mental health and higher quality of life. While income was significantly associated with four out of six health indicators in model 1, all associations became insignificant after introducing financial hardship as control variable in model 2. However, financial hardship was gradually linked to all six health indicators in the expected direction (p value in all cases <0.001).

**Table 3:** Associations of socioeconomic circumstances with health indicators, adjusted odds ratios of ordinal logistic regressions and (95% confidence intervals).

	Secondary conditions	Comorbidities	Pain intensity	Mental health	Participation	Quality of life
[N]	[1190]	[1460]	[1481]	[1362]	[1491]	[1499]
**Education in years**						
Model 1	1.00 (0.96–1.04)	0.98 (0.94–1.03)	0.99 (0.96–1.02)	1.03 (1.00–1.06)	1.02 (0.99–1.06)	1.05 (1.01–1.09)
*p* value	*0.952*	*0.455*	*0.652*	*0.093*	*0.130*	*0.006*
Model 2	0.99 (0.96–1.03)	0.98 (0.94–1.02)	0.99 (0.96–1.02)	1.04 (1.00–1.07)	1.03 (1.00–1.06)	1.06 (1.02–1.09)
*p* value	*0.698*	*0.265*	*0.478*	*0.039*	*0.078*	0.002
**Net equivalence household income (units of 1000 Swiss Francs)**						
Model 1	0.96 (0.90–1.02)	0.92 (0.85–0.99)	0.90 (0.85–0.95)	1.10 (1.03–1.16)	1.04 (0.99–1.10)	1.15 (1.09–1.23)
*p* value	*0.245*	*0.035*	*<.001*	*0.002*	*0.130*	*<.001*
Model 2	1.03 (0.97–1.11)	0.98 (0.91–1.07)	0.96 (0.90–1.01)	1.01 (0.95–1.07)	0.98 (0.92–1.03)	1.06 (0.99–1.13)
*p* value	*0.306*	*0.685*	*0.135*	*0.729*	*0.408*	*0.085*
**Financial hardship (model 1)**						
No difficulties	Ref.	Ref.	Ref.	Ref.	Ref.	Ref.
Some difficulties	2.07 (1.56–2.75)	1.78 (1.31–2.43)	1.60 (1.25–2.06)	0.44 (0.34–0.57)	0.59 (0.46–0.76)	0.43 (0.33–0.65)
Severe difficulties	3.37 (2.18–5.21)	2.88 (1.83–4.53)	3.32 (2.21–4.99)	0.23 (0.15–0.36)	0.30 (0.21–0.43)	0.22 (0.15–0.33)
*p* value	*<.001*	*<.001*	*<.001*	*<.001*	*<.001*	*<.001*

*p* values are from equal fraction-missing-information (FMI) test.

Note: Predictors and covariates imputed by multiple imputation, full case outcomes in this table. Results are weighted by propensity scores.

Model 1: adjusted for age, gender, lesion characteristics (para/tetraplegia, completeness of injury, years since injury), aetiology, social support, education, and income.

Model 2: Model 1 + financial hardship.

## Discussion

This study of a large cohort of Swiss men and women with SCI demonstrates consistent associations of financial hardship with all health indicators under study. Experiencing severe financial difficulties goes along with more health problems as well as reduced functioning and quality of life. This holds true even for those reporting only some financial difficulties. Interestingly, the links with established indicators of social inequalities are weaker in case of education or non-existent in case of household income when adjusting for perceived financial hardship.

In light of these inconsistent associations, the question arises why financial hardship exerts such strong and consistent effects on health. One possible argument is that financial difficulties aggravate people's burden of everyday life as they restrict the access to relevant resources and generate feelings of relative deprivation with subsequent stress reactions [13], [45], [46]. Thus, suffering from financial hardship might reflect material disadvantage as well as psychosocial stress, e.g. due to restricted access to other health-relevant resources such as social capital, or health care. Alternatively, one could argue that people's financial hardship results from their health problems. This argument cannot be excluded in the frame of a cross-sectional study design. However, we found little evidence in its favor as 1) we found no association between lesion level and financial hardship, and 2) the association between financial hardship and health was not modified after introducing variables on the severity of disability into multivariable models.

The link between education and health was restricted to mental health and quality of life when financial hardship was considered in the same model and was therefore less consistent as compared to the one observed with financial hardship. In case of income, none of the associations was significant after adjustment for all other influence factors and potential confounders. Whether these findings can be replicated or whether they reflect imprecise measures in populations with disabilities needs to be analysed in further studies. For instance, as education was measured by a summary index of school and vocational training, it may not adequately reflect the socioeconomic circumstances of persons with SCI, particularly as many of them underwent long-standing vocational re-training. A similar point can be made for household income. First, household income only moderately correlates with financial hardship (Pearson correlation coefficient −0.335) thus indicating somewhat different economic facts. Second, net equivalence household income may not adequately take into account the economic burden of people with disabilities, given their regular additional health care expenditures or other fixed costs related to the disabling condition (e.g. special equipment, assistive devices).

Yet, in line with our main finding of a consistent association of financial hardship with health, a recent cohort study demonstrated a strong effect of financial hardship on mortality, above and beyond the one produced by education and income [47]. It is noteworthy that financial difficulties are related to impaired health even in one of the wealthiest countries worldwide which offers far-reaching health services and social security policies to people with disability.

### Strengths and limitations

This study has several limitations. First, findings are based on cross-sectional data, which does not allow to draw conclusions on causal associations. Second, our sample may not be fully representative of persons with an SCI living in Switzerland. Although several SCI-clinics and the national association for persons with SCI have been involved in recruitment [24], the exact number of persons with an SCI living in Switzerland is unknown. Moreover, the robustness of reported findings needs further exploration and the clinical significance of observed differences according to socioeconomic circumstances needs to be elaborated.

Despite these limitations, our study has several strengths. The study population is based on a large community sample rather than on some selected group, and data meet high quality standards. We analyse a comprehensive set of health indicators thus drawing a detailed picture on health problems, functioning and quality of life in persons with SCI. Moreover, we conducted additional sensitivity analyses to correct for item and unit nonresponse. Given the small number of aberrations (see Table S1), we conclude that our findings are robust against bias due to missing values and nonresponse.

## Conclusion

In this study on the association of socioeconomic circumstances with health in persons with disabilities, financial hardship was the only indicator consistently related to reduced health. This indicator may reflect material as well as psychosocial adversity in people with disabilities. As this association was evident in Switzerland, one of the wealthiest countries worldwide, even stronger effects of financial hardship on health might be expected in less developed countries. If supported by further research, our results may have implications for more equitable resource allocation.

## Supporting Information

Appendix S1 **Details of the imputation modeling.**
(DOCX)

Table S1 **Sensitivity analyses of associations between socioeconomic circumstances and health indicators, odds ratios of ordinal regressions and (95% confidence intervals) using different scenarios to handle missing values, weighted (W) and unweighted (U) results.**
(DOCX)
