# Supplementary material for: Is Financial Hardship Associated with Reduced Health in Disability? The Case of Spinal Cord Injury in Switzerland
Source: PLoS One. 2014 Feb 28;9(2):e90130. doi: 10.1371/journal.pone.0090130 (PMC3938582; doi:10.1371/journal.pone.0090130)
Supplement: Appendix S1 — Details of the imputation modeling. (DOCX) [file pone.0090130.s001.docx]

## **Appendix S1:** Details of the imputation modeling

## We used the ‘mi impute chained’ command of Stata Version 13 for the imputation modeling. This command uses a chained-equation algorithm to impute missing values in multiple variables based on an iterative sampling process. Imputations were carried out for 10 datasets and we used the following imputation procedures for variables that contained missing values (see Table 1 for the number of missing values for each variable):

## Dichotomous variables (partner status, living arrangement, lesion level, completeness of lesion, aetiology): logistic regression (logit)

## Continuous variables (number of children, relevant to weight household income): Poisson regression (poisson)

## Continuous variables (years since injury, education in years): Truncated regression (truncreg)

## Ordinal variables (financial hardship, household income): Ordered logistic regression (ologit)

## Age and gender had no missing values

## Outcome parameters were not imputed.
