# Supplementary material for: Is Financial Hardship Associated with Reduced Health in Disability? The Case of Spinal Cord Injury in Switzerland
Source: PLoS One. 2014 Feb 28;9(2):e90130. doi: 10.1371/journal.pone.0090130 (PMC3938582; doi:10.1371/journal.pone.0090130)
Supplement: Table S1 — Sensitivity analyses of associations between socioeconomic circumstances and health indicators, odds ratios of ordinal regressions and (95% confidence intervals) using different scenarios to handle missing values, weighted (W) and unweighted (U) results. (DOCX) [file pone.0090130.s002.docx]

## **Table S1:** Sensitivity analyses of associations between socioeconomic circumstances and health indicators, odds ratios of ordinal regressions and (95% confidence intervals) using different scenarios to handle missing values, weighted (W) and unweighted (U) results

| [N] |  | Education | *p* | Income | *p* | Some financial difficulties | Severe financial difficulties | *p* |
| --- | --- | --- | --- | --- | --- | --- | --- | --- |
| **Secondary conditions** | | | |  |  |  |  |  |
| Scenario 1 [1190] | U | 0.99 (0.96-1.02) | 0.725 | 1.03 (0.97-1.10) | 0.369 | 1.99 (1.51-2.61) | 3.25 (2.15-4.90) | <0.001 |
|  | W | 0.99 (0.96-1.03) | 0.698 | 1.03 (0.97-1.11) | 0.306 | 2.07 (1.56-2.75) | 3.37 (2.18-5.21) | <0.001 |
| Scenario 2 [1042] | U | 0.99 (0.96-1.03) | 0.693 | 1.02 (0.96-1.10) | 0.471 | 2.05 (1.54-2.72) | 3.47 (2.22-5.42) | <0.001 |
|  | W | 0.99 (0.95-1.03) | 0.683 | 1.03 (0.96-1.11) | 0.379 | 2.16 (1.61-2.90) | 3.63 (2.25-5.84) | <0.001 |
| Scenario 3 [1549] | U | 1.02 (0.99-1.05) | 0.221 | 1.03 (0.97-1.09) | 0.306 | 1.76 (1.39-2.24) | 2.16 (1.49-3.15) | <0.001 |
|  | W | 1.02 (0.99-1.05) | 0.217 | 1.03 (0.97-1.10) | 0.272 | 1.82 (1.41-2.33) | 2.19 (1.45-3.33) | <0.001 |
| Scenario 4 [1549] | U | 0.98 (0.96-1.01) | 0.307 | 0.96 (0.91-1.02) | 0.156 | 1.73 (1.37-2.19) | 2.92 (2.00-4.26) | <0.001 |
|  | W | 0.99 (0.95-1.02) | 0.374 | 0.97 (0.91-1.03) | 0.283 | 1.83 (1.44-2.34) | 3.02 (2.03-4.50) | <0.001 |
| **Comorbidities** | | | |  |  |  |  |  |
| Scenario 1 [1460] | U | 0.98 (0.94-1.02) | 0.230 | 0.99 (0.91-1.06) | 0.714 | 1.76 (1.31-2.37) | 3.07 (2.02-4.68) | <0.001 |
|  | W | 0.98 (0.94-1.02) | 0.265 | 0.98 (0.91-1.07) | 0.685 | 1.78 (1.31-2.43) | 2.88 (1.83-4.53) | <0.001 |
| Scenario 2 [1244] | U | 0.98 (0.94-1.01) | 0.267 | 0.97 (0.90-1.05) | 0.509 | 1.73 (1.26-2.36) | 3.04 (1.92-4.82) | <0.001 |
|  | W | 0.98 (0.94-1.02) | 0.360 | 0.97 (0.89-1.05) | 0.441 | 1.75 (1.28-2.41) | 2.84 (1.71-4.71) | <0.001 |
| Scenario 3 [1549] | U | 0.99 (0.96-1.03) | 0.761 | 0.98 (0.91-1.06) | 0.672 | 1.68 (1.26-2.25) | 2.58 (1.73-3.88) | <0.001 |
|  | W | 1.00 (0.96-1.03) | 0.947 | 0.98 (0.91-1.06) | 0.585 | 1.70 (1.26-2.29) | 2.40 (1.54-3.74) | <0.001 |
| Scenario 4 [1549] | U | **0.96 (0.93-1.00)** | **0.046** | 0.97 (0.91-1.05) | 0.475 | 1.76 (1.33-2.35) | 3.01 (2.02-4.49) | <0.001 |
|  | W | 0.96 (0.93-1.01) | 0.087 | 0.97 (0.90-1.05) | 0.447 | 1.79 (1.32-2.41) | 2.83 (1.87-4.30) | <0.001 |
| **Pain intensity** | | | |  |  |  |  |  |
| Scenario 1 [1481] | U | 0.99 (0.96-1.08) | 0.404 | 0.96 (0.99-1.01) | 0.141 | 1.51 (1.18-1.92) | 3.12 (2.14-4.56) | <0.001 |
|  | W | 0.99 (0.96-1.02) | 0.478 | 0.96 (0.90-1.01) | 0.135 | 1.60 (1.25-2.06) | 3.32 (2.21-4.99) | <0.001 |
| Scenario 2 [1268] | U | 0.99 (0.96-1.02) | 0.405 | 0.96 (0.90-1.02) | 0.251 | 1.47 (1.14-1.89) | 3.82 (2.52-5.78) | <0.001 |
|  | W | 0.99 (0.96-1.03) | 0.614 | 0.96 (0.91-1.02) | 0.237 | 1.59 (1.22-3.07) | 4.10 (2.60-6.46) | <0.001 |
| Scenario 3 [1549] | U | 0.99 (0.96-1.02) | 0.608 | 0.97 (0.92-1.02) | 0.261 | 1.49 (1.17-1.90) | 2.95 (2.03-4.28) | <0.001 |
|  | W | 0.99 (0.96-1.03) | 0.746 | 0.97 (0.92-1.03) | 0.291 | 1.57 (1.22-2.03) | 3.01 (1.99-4.58) | <0.001 |
| Scenario 4 [1549] | U | 0.99 (0.96-1.02) | 0.386 | 0.95 (0.90-1.01) | 0.098 | 1.44 (1.13-1.83) | 2.89 (1.98-4.22) | <0.001 |
|  | W | 0.99 (0.96-1.02) | 0.437 | 0.95 (0.90-1.01) | 0.095 | 1.52 (1.19-1.95) | 3.08 (2.06-4.59) | <0.001 |
| **Mental health** | | | |  |  |  |  |  |
| Scenario 1 [1362] | U | 1.03 (1.00-1.07) | 0.045 | 1.01 (0.95-1.07) | 0.797 | 0.45 (0.35-0.58) | 0.24 (0.16-0.35) | <0.001 |
|  | W | 1.04 (1.00-1.07) | 0.039 | 1.01 (0.95-1.07) | 0.729 | 0.44 (0.34-0.57) | 0.23 (0.15-0.36) | <0.001 |
| Scenario 2 [1185] | U | 1.04 (1.01-1.08) | 0.018 | 1.00 (0.94-1.06) | 0.982 | 0.45 (0.34-0.58) | 0.21 (0.14-0.33) | <0.001 |
|  | W | 1.04 (1.01-1.08) | 0.016 | 1.00 (0.94-1.07) | 0.958 | 0.44 (0.34-0.57) | 0.21 (0.13-0.34) | <0.001 |
| Scenario 3 [1549] | U | **1.02 (1.00-1.06)** | **0.093** | 1.01 (0.95-1.06) | 0.850 | 0.49 (0.39-0.62) | 0.27 (0.19-0.38) | <0.001 |
|  | W | **1.03 (1.00-1.06)** | **0.075** | 1.01 (0.95-1.07) | 0.737 | 0.48 (0.38-0.62) | 0.26 (0.17-0.39) | <0.001 |
| Scenario 4 [1549] | U | 1.03 (1.00-1.06) | 0.040 | 1.02 (0.97-1.09) | 0.370 | 0.46 (0.36-0.59) | 0.29 (0.20-0.42) | <0.001 |
|  | W | 1.04 (1.00-1.93) | 0.032 | 1.02 (0.96-1.09) | 0.447 | 0.46 (0.36-0.89) | 0.29 (0.19-0.44) | <0.001 |
| **Participation** | | |  |  |  |  |  |  |
| Scenario 1 [1491] | U | 1.03 (1.00-1.06) | 0.080 | 0.99 (0.94-1.05) | 0.762 | 0.61 (0.48-0.78) | 0.31 (0.22-0.45) | <0.001 |
|  | W | 1.03 (1.00-1.06) | 0.078 | 0.98 (0.92-1.03) | 0.408 | 0.59 (0.46-0.76) | 0.30 (0.21-0.43) | <0.001 |
| Scenario 2 [1276] | U | 1.02 (0.99-1.06) | 0.107 | 0.98 (0.92-1.05) | 0.549 | 0.64 (0.49-0.82) | 0.29 (0.19-0.42) | <0.001 |
|  | W | 1.03 (1.00-1.07) | 0.085 | 0.97 (0.91-1.03) | 0.280 | 0.61 (0.48-0.80) | 0.27 (0.19-0.39) | <0.001 |
| Scenario 3 [1536]+ | U | 1.01 (0.98-1.05) | 0.338 | 0.98 (0.93-1.04) | 0.596 | 0.60 (0.93-1.04) | 0.31 (0.21-0.44) | <0.001 |
|  | W | 1.02 (0.98-1.05) | 0.322 | 0.97 (0.92-1.03) | 0.365 | 0.58 (0.45-0.74) | 0.29 (0.20-0.43) | <0.001 |
| Scenario 4 [1536]+ | U | **1.04 (1.01-1.08)** | **0.008** | 1.04 (0.98-1.11) | 0.224 | 0.62 (0.49-0.80) | 0.35 (0.25-0.51) | <0.001 |
|  | W | **1.04 (1.01-1.08)** | **0.010** | 1.02 (0.96-1.10) | 0.416 | 0.62 (0.47-0.81) | 0.34 (0.24-0.48) | <0.001 |
| +13 cases with completely missing scales excluded | | | |  |  |  |  | <0.001 |
| **Quality of life** | | |  |  |  |  |  |  |
| Scenario 1 [1499] | U | 1.05 (1.02-1.09) | 0.002 | 1.05 (0.99-1.12) | 0.099 | 0.45 (0.35-0.58) | 0.22 (0.16-0.32) | <0.001 |
|  | W | 1.06 (1.02-1.09) | 0.002 | 1.06 (0.99-1.13) | 0.085 | 0.43 (0.33-0.65) | 0.22 (0.15-0.33) | <0.001 |
| Scenario 2 [1276] | U | 1.06 (1.03-1.10) | 0.001 | 1.04 (0.98-1.11) | 0.204 | 0.41 (0.32-0.54) | 0.19 (0.13-0.29) | <0.001 |
|  | W | 1.07 (1.03-1.10) | <0.001 | 1.04 (0.97-1.11) | 0.250 | 0.40 (0.31-0.53) | 0.19 (0.12-0.29) | <0.001 |
| Scenario 3 [1549] | U | 1.04 (1.01-1.08) | 0.005 | 1.04 (0.98-1.10) | 0.243 | 0.48 (0.37-0.62) | 0.23 (0.16-0.34) | <0.001 |
|  | W | 1.05 (1.01-1.08) | 0.005 | 1.04 (0.98-1.11) | 0.225 | 0.47 (0.36-0.62) | 0.24 (0.16-0.36) | <0.001 |
| Scenario 4 [1549] | U | 1.05 (1.02-1.09) | 0.002 | 1.06 (0.99-1.13) | 0.053 | 0.45 (0.35-0.59) | 0.27 (0.18-0.39) | <0.001 |
|  | W | 1.05 (1.02-1.08) | 0.003 | 1.06 (1.00-1.12) | 0.055 | 0.46 (0.36-0.60) | 0.26 (0.19-0.38) | <0.001 |

## For scenario 1, 3, 4 (weighted and unweighted): *p* values from equal fraction-missing-information (FMI) test; for scenario 2 (unweighted): *p* values from likelihood-ratio test; for scenario 2 (weighted): *p* values from Wald-test.

## Scenario 1: Imputed values for socioeconomic circumstances/controls, health indicators only full cases.

## Scenario 2: Only full cases in all included variables.

## Scenario 3: Imputed values for socioeconomic circumstances/controls, health indicators replaced by ‘best case’.

## Scenario 4: Imputed values for socioeconomic circumstances/controls, health indicators replaced by ‘worst case’.

## All models are adjusted for age, gender, lesion characteristics (para/tetraplegia, completeness of injury, years since injury), aetiology, social support, and socioeconomic circumstances.

## **Results** printed in bold indicate aberrations from the weighted scenario 1 (results shown in Table 3).
